# Omental torsion: diagnostic challenge in patients with sclerosing cholangitis and inflammatory bowel disease – case report

**DOI:** 10.1097/MS9.0000000000001049

**Published:** 2023-07-08

**Authors:** Neha Malik, Saziini H. Chorosho, Pratima Kumari, Rupinder Kaur, Ravinder Singh, Hitesh Chopra, Talha Bin Emran

**Affiliations:** a Chitkara College of Pharmacy, Chitkara University, Punjab, India; b Department of Pharmacy, BGC Trust University Bangladesh, Chittagong, Bangladesh; c Department of Pharmacy, Faculty of Allied Health Sciences, Daffodil International University, Dhaka, Bangladesh

**Keywords:** case report, inflammatory bowel disease, laparoscopy, omental torsion, sclerosing cholangitis

## Abstract

**Introduction and importance:**

The omentum appears as an apron-like extension of the peritoneum.

**Case presentation:**

A 30-year-old male patient, presented to the emergency department with the chief complaints of acute nonradiating pain localized in the right-side abdomen for the past 3 days. The patient had a past medical history of sclerosing cholangitis (SC) with inflammatory bowel disease (IBD). The patient reported the pain as persistent, pressure-like, and moderate. The patient also had a low-grade fever and nausea at the time of admission. On examination, the vital signs were found as normal. The patient reported that the abdominal pain gets exacerbated after the meals, and increase in physical activity and movement. Due to the patient’s complaints and history of SC and IBD, these were considered as the possible diagnosis. After the diagnostic procedures, the patient was finally diagnosed with OT.

**Clinical discussion:**

This report presents a case of a patient suffering from omental torsion having history of SC and IBD. During the laparoscopic procedure, the diagnosis of omental torsion was confirmed. To our knowledge, no case report of omental torsion with IBD and SC is published in the literature.

**Conclusion:**

This seems to be a major diagnostic challenge as patients with IBD almost resembles the same clinical signs and symptoms as in the omental torsion. The possibility of misdiagnosis and delayed diagnosis could result in the unfavorable outcome. Therefore, the healthcare fraternities are advised to include the rare diseases such as OT as the differential diagnosis.

## Background

Omental torsion is a rare condition characterized by acute pain in the abdomen. This condition can either be primary or secondary. This condition predominately affects males than females and more prevalent in adults. The exact etiology of the condition is unknown. However, it is associated with several risk factors such as obesity, cysts, hernias, and tumors. This condition can be managed conservatively with antibiotics, analgesics and anti-inflammatory agents. However, if the symptoms did not improve or the condition deteriorates (such as infarcted omentum) then definite treatment should be used. Laparotomy is performed as the definite treatment of OT and it involves resection of the torted or infarcted omentum. Clinically, patients with omental torsion presents with flank abdominal pain, frequently localized in the right-side of the abdomen, low-grade fever, nausea, and vomiting. OT mimics a number of other disease conditions such as acute appendicitis, cholecystitis, intestinal obstruction, and inflammatory bowel disease. These findings are nonspecific and can lead to misdiagnosis. The diagnosis of OT is generally made by ruling out the other disease conditions such as appendicitis and cholecystitis. The confirmatory diagnosis can be done abdominal ultrasound or CT scan. The nonspecific characteristics of condition, misdiagnosis, and delayed diagnosis pose the threat to patient’s health.

### Introduction

Highlights

- The omentum appears as an apron-like extension of the peritoneum.
- The rotation on the long axis of the omentum is known as omental torsion.
- Ultrasonography or computed tomography scan remains a primary diagnostic tool.

The omentum appears as an apron-like extension of the peritoneum, originating from the abdomen, and then descending towards the anterior part of the small intestine, further ascending and getting attached to the transverse colon^1,2^. The rotation on the long axis of the omentum or twisting of omentum is known as OT. OT results in vascularity compromise on the distal parts of the omentum leading to tissue infarction and could result into infarcted omentum^3^.

OT occurs in a right-handed or clockwise direction where the venous blood return is affected resulting in congestion as well as edematous of the distal omentum. The etiology of OT is not exactly clear. There are various predisposing and precipitating factors reported for OT. Predisposing factors include the anatomical and vascular anamolies such as the expressions of tongue-like projections bifid omentum, abnormal vascular blood supply, trauma, obesity, and hyperperistalsis that further leads to variation in the weight of the omentum and displacement of the omentum. The precipitating factors includes cough, sudden movements or change in the posture of body like in the case of increased bowel motion, occupational hazards with vibrating tools and conditions that induce displacement in the omentum, elevated intra-abdominal strain, as a consequence of heavy exercise, sneezing, whooping, or excess straining^4–8^.

The primary omental torsion (POT) is a mobile and thick omentum segment rotating on a fixed point proximally with no secondary intra-abdominal pathology. The various factors associated with variation in the anatomy of the primary omentum include tongue-like extensions projecting through the free edge, accessory omentum, bifid omentum, obesity resulting in the immoderate deposition of fat tissue inside the omentum, and a contracted omental pedicle. POT and secondary omental torsion (SOT) both presents similar clinical characteristics. The most typical symptom is a sudden onset of pain in the right iliac fossa in association with vomiting and nausea. When there is no underlying cause for the condition, it is known as POT. On the other hand, SOT is linked to other intra-abdominal pathologies such as omental cysts, adhesions, hernias, and tumors^9^.

Ultrasonography or computed tomography (CT) scan remains a primary diagnostic tool as it helps for the diagnosis of OT avoiding the chances of misdiagnosis^10^. MRI can also be useful when OT is accompanied by complications such as the growth of an abscess or excessive bleeding.

Laparoscopy is considered as a confirmatory tool for the diagnosis of OT. At laparoscopy, free serosanguineous fluid can be seen in the abdomen^11–13^.

Omental torsion is usually misdiagnosed as it mimics a number of other conditions such as cholecystitis, acute appendicitis, intestinal obstruction, perforated duodenal ulcer, and abdominal wall hematoma. The diagnosis of omental torsion in patients with inflammatory bowel disease (IBD) and PSC remains a major challenge as all these clinical conditions present the common symptoms such as abdominal pain, low-grade fever, nausea, and vomiting that are nonspecific to the condition. Hence, this condition can be misdiagnosed as acute appendicitis, IBD and cholecystitis, which may results in the unfavorable outcomes.

## Case report

A 30-year-old male patient, presented to the emergency department with the chief complaint of acute nonradiating pain localized in the right-side abdomen for the past 3 days. The patient had a past medical history of sclerosing cholangitis (SC) with IBD. The patient reported the pain as persistent, pressure-like, and moderate. The patient also had a low-grade fever and nausea at the time of admission. On examination, the vital signs were found as normal. The patient reported that the abdominal pain gets exacerbated after the meals, and increase in physical activity and movement.

**Figure 1:**
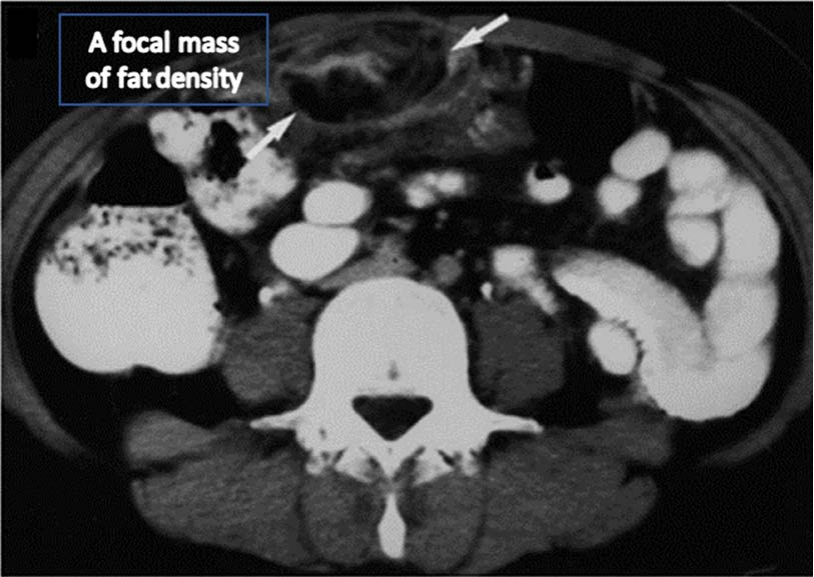
Computed tomography section of the abdomen showing a focal mass of fat density (indicated with white arrows).

On examination, the abdomen was found to be soft and slightly distended. Besides, tenderness was observed in the upper quadrant and iliac fossa specifically on the right-side, as well as physiological signs of the discomfort in the peritoneum. A complete blood count showed moderate leukocytosis. A CT scan was conducted to examine the appendix as the patient presented with appendicitis-like symptoms^14^. However, the test indicated a normal appendix with no strands. The test also revealed the appearance of thickened fat tissues, oval in structure along with twisting of blood vessels in the right abdomen (Fig. 1).

An radiograph of the chest showed no air below the diaphragm. Clinical findings of renal and hepatic function tests were at normal levels and serum amylase was found to be 105 U/l. Examination of the hernial orifices, genitalia, rectum, prostate and the pelvis were found to be normal and the rectum was empty.

**Figure 2:**
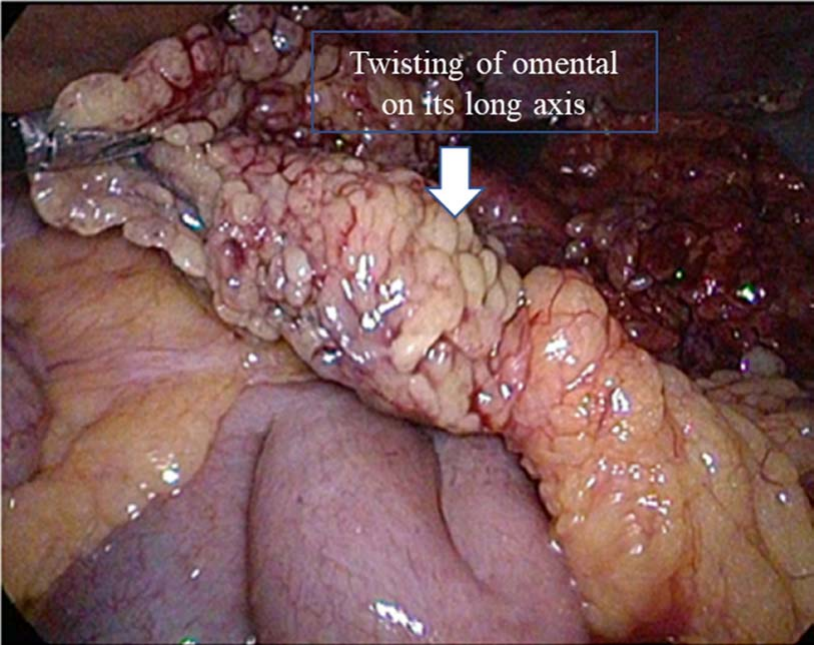
Laparoscopic image of torted omentum.

Laparoscopy revealed a little amount of blood with some inflammatory mass and a part of the omentum that is infarcted. It also revealed that a segment of the greater omentum to have been torted several times around a narrow base. It confirmed a hemorrhagic infarction with necrosis into the greater omentum, connected to the proximal transverse colon, while the remaining parts were normal (Fig. 2).

## Discussion

Due to the patient’s complaints and history of SC and IBD, these were considered as the possible diagnosis. It was assumed that the abdominal pain was caused by IBD spasms, but the CT scan results indicate that the patient has OT. The torsion in the greater omentum is uncommon, accounting for around 1.1% of overall cases of acute abdominal pain. The most typical sign of POT is an abrupt onset of abdominal discomfort or pain, specifically in the right lower quadrant, and generally nonradiating. Short-duration episodes of vomiting, nausea as well as a slightly elevated body temperature may occur along with the abdominal pain^15^. The SOT is commonly in association with susceptible pathologies such as hernial sacs, surgical scars or wounds, tumors, and cysts. However, inguinal hernia is considered the most prevalent pathological factor^16^. Another cause is the venous redundancy relating to the arterial blood supply in the omentum, which permits venous bending as well as provides a fixed point for the omental segment to twist around^4^. Both types of OT share common characteristics but are distinguished in the fashion that POT is unipolar, while the SOT can be both unipolar and bipolar. The unipolar OT involves a fixed proximal omentum and the other tongues remain free. On the other hand, in bipolar OT, both the proximal and distal omenta are fixed in place^17^. However, SOT is more prevalent than the primary type.

The occurrence of omental torsion on the left side is very rare because the right part of the omentum is heavier, mobile, and longer as compared to the left side. The most frequent presentation is a single instance of flank pain, with recurrence indicating intermittent torsions^18^. The pathology of OT includes rotation of its long axis, which causes the diminishing of the vasculature resulting in impairment in blood supply. When the omental torsion exacerbates, the arterial occlusion occurs and results in acute hemorrhagic infarction, which leads to necrosis of the omentum tissues known as infarcted omentum.

OT mimics some different acute abdominal conditions, for instance, cholecystitis, acute appendicitis, intestinal obstruction, perforated duodenal ulcer, and abdominal wall hematoma. Besides, females of child bearing age should be considered with conditions such as salpingitis, ovarian cyst torsion, and ectopic pregnancy^19^.

Ultrasonography, CT scan, laparoscopy and MRI can be used for the diagnosis of OT. Once diagnosed, initially the condition can be managed conservatively with antibiotics, analgesics, and anti-inflammatory agents. However, if the symptoms did not improve or the condition deteriorates (such as infarcted omentum) then a definite treatment should be used. Laparotomy can be performed as the definite treatment of OT and it involves resection of the torted or infarcted omentum. The benefits of laparoscopy involve a detailed evaluation of the abdomen for confirming the diagnosis, as well as the advantages of minimal-access surgery, such as the reduction of postoperative pain as well as wound-related complications. The conservative management of OT is the rational alternative to surgery for hemodynamically stable patients^20^. Patients who are conservatively treated may require prolonged analgesics, anti-inflammatory agents and antibiotic treatment. The prolonged treatment can cause the complication such as the formation of abscesses and adhesions, which are precipitated in the abdomen region because of the presence of necrotic tissue. Ethical approval for this case report has been reported in line with the Surgical Case Report (SCARE) criteria. The patient provided permission for the case to be published for scholarly and research purposes.

## Conclusion

This report presents a case of a patient suffering from omental torsion having history of SC and IBD. Omental torsion is a rare phenomenon that is often misdiagnosed with acute appendicitis and cholecystitis. Omental torsion is frequently misdiagnosed because it resembles cholecystitis, acute appendicitis, intestinal blockage, ruptured duodenal ulcer, and abdominal wall hemorrhage. The diagnosis of omental torsion in patients with IBD and PSC remains difficult since all of these clinical disorders present with nonspecific symptoms such as stomach discomfort, low-grade fever, nausea, and vomiting. As a result, this condition may be misinterpreted as acute appendicitis, IBD, or cholecystitis, leading to the unfavorable outcomes. Therefore, the healthcare fraternities are advised to include the rare diseases such as OT as the differential diagnosis.

## Ethical approval

This case report has been reported in line with the SCARE criteria.

## Consent

Written informed consent was obtained from the patient for the publication of this case report and accompanying images. A copy of the written consent is available for review by the Editor-in-Chief of this journal on request.

## Sources of funding

None.

## Author contribution

N.M., S.H.C., P.K., R.K., R.S., H.C.: conceptualization, data curation, writing – original draft preparation, writing – reviewing and editing; T.B.E.: conceptualization, writing – reviewing and editing, visualization.

## Conflicts of interest disclosure

The authors declare that they have no financial conflict of interest with regard to the content of this report.

## Research registration unique identifying number (UIN)

1. Name of the registry: not applicable.
2. Unique Identifying number or registration ID: not applicable.
3. Hyperlink to your specific registration (must be publicly accessible and will be checked): not applicable.

## Gurantor

Talha Bin Emran.

## Data availability statement

All data available in this manuscript.

## Provenance and peer review

Not commissioned, externally peer-reviewed.
